# Endothelium-Derived Dopamine and 6-Nitrodopamine in the Cardiovascular System

**DOI:** 10.1152/physiol.00020.2023

**Published:** 2023-10-24

**Authors:** Roberto Zatz, Gilberto De Nucci

**Affiliations:** ^1^Renal Division, Department of Clinical Medicine, Faculty of Medicine, University of São Paulo, São Paulo, Brazil; ^2^Department of Pharmacology, Faculty of Medical Sciences, State University of Campinas (UNICAMP), Campinas, São Paulo, Brazil; ^3^Department of Pharmacology, Institute of Biomedical Sciences, University of São Paulo (ICB-USP), São Paulo, Brazil; ^4^Department of Pharmacology, Faculty of Medicine, São Leopoldo Mandic, Campinas, São Paulo, Brazil; ^5^Department of Pharmacology, Faculty of Medicine, Metropolitan University of Santos, Santos, São Paulo, Brazil

**Keywords:** chronotropism, 6-cyanodopamine, inotropism, nitric oxide, nitrocatecholamine

## Abstract

The review deals with the release of endothelium-derived dopamine and 6-nitrodopamine (6-ND) and its effects on isolated vascular tissues and isolated hearts. Basal release of both dopamine and 6-ND is present in human isolated umbilical cord vessels, human popliteal vessels, nonhuman primate vessels, and reptilia aortas. The 6-ND basal release was significantly reduced when the tissues were treated with *N*^ω^-nitro-l-arginine methyl ester and virtually abolished when the endothelium was mechanically removed. 6-Nitrodopamine is a potent vasodilator, and the mechanism of action responsible for this effect is the antagonism of dopamine D_2_-like receptors. As a vasodilator, 6-ND constitutes a novel mechanism by which nitric oxide modulates vascular tone. The basal release of 6-ND was substantially decreased in endothelial nitric oxide synthase knockout (eNOS^−/−^) mice and not altered in neuronal nitric oxide synthase knockout (nNOS^−/−^) mice, indicating a nonneurogenic source for 6-ND in the heart. Indeed, in rat isolated right atrium, the release of 6-ND was not affected when the atria were treated with tetrodotoxin. In the rat isolated right atrium, 6-ND is the most potent endogenous positive chronotropic agent, and in Langendorff’s heart preparation, it is the most potent endogenous positive inotropic agent. The positive chronotropic and inotropic effects of 6-ND are antagonized by β_1_-adrenoceptor antagonists at concentrations that do not affect the effects induced by noradrenaline, adrenaline, and dopamine, indicating that blockade of the 6-ND receptor is the major modulator of heart chronotropism and inotropism. The review proposes that endothelium-derived catecholamines may constitute a major mechanism for control of vascular tone and heart functions, in contrast to the overrated role attributed to the autonomic nervous system.

## Introduction

The autonomic nervous system is believed to be a major regulator in the cardiovascular system and its adaptation to various human body functions (1). Both adrenergic and cholinergic innervation are present in the heart, and the cardiac effect of the autonomic nervous system depends on the balance of these two antagonistic sets of nerves. Most of the data concerning the role of adrenergic innervation in the cardiovascular system have been derived from two basic assumptions: catecholamines are released mainly from adrenergic nerve terminals and the belief that drugs such as propranolol (2), atenolol (3), and metoprolol (3) are acting either as β_1_β_2_ in the case of propranolol or β_1_-selective adrenoceptor antagonists in the case of the others. Another important modulator of the cardiovascular system is the endothelium-derived relaxing factor discovered by Furchgott and Zawadzki (4), later identified as nitric oxide (5, 6). Nitric oxide causes vasodilatation (7), lowers blood pressure (8), inhibits platelet adhesion (9), and reduces smooth muscle proliferation (10). Nitrergic neurogenic vasodilation was demonstrated when cerebral arteries relaxed in response to electrical stimulation (11, 12). Precontracted mammalian corpus cavernosum relaxes following electric field stimulation, and this relaxation is inhibited by pretreatment of the tissue with inhibitors of nitric oxide (NO) synthase such as *N*^ω^-nitro-l-arginine methyl ester (l-NAME) or the voltage-gated sodium channel blocker tetrodotoxin (14).

The first discrepancy noted in the rationale described above occurred when the relaxation of *Crotalus durissus terrificus* (rattlesnake) precontracted corpus cavernosum (induced by electric field stimulation) was investigated. Although the relaxation was inhibited by l-NAME, it was not affected by pretreatment with tetrodotoxin (14). Indeed, in noncontracted corpus cavernosum, electrical field stimulation caused contractions of the rattlesnake corpus cavernosum and aortic rings, which were insensitive to pretreatment with tetrodotoxin (13, 15).

This review will focus on the release of dopamine and 6-nitrodopamine by the endothelium and their role in the cardiovascular system. The data indicate that nonneurogenic catecholamines play a fundamental role in the modulation of the cardiovascular system and the results obtained with the so-called β-blockers were misinterpreted, generating an overestimation of the role of the adrenergic nervous system.

The first evidence that endothelial cells could synthesize catecholamines was described in cultured bovine aortic endothelial cells (BAECs), where the presence of the enzymes involved in the catecholamine biosynthetic pathway such as tyrosine hydroxylase, was detected when the cells were submitted to hypoxia (16). Release of noradrenaline and adrenaline was also detected by an ELISA in the culture medium of the BAECs in both normoxic and hypoxic conditions (16).

The first indirect evidence that endothelial-derived catecholamines could modulate vascular smooth muscle tonus was observed in isolated aortic rings obtained from the snake *Pantherophis guttatus* (corn snake) that were contracted by electrical field stimulation (17). Preincubation of the aortic rings with the inhibitor of the voltage-gated sodium channel tetrodotoxin failed to affect the contractions; however, they were significantly reduced by the adrenoceptor blockers such as phentolamine and virtually abolished when the endothelium was mechanically removed (17). Similar results were obtained with aortic rings of the venomous snakes *Bothrops jararaca* and rattlesnake (15) and in the aortic rings of the tortoise *Chelonoidis carbonaria* (18), where immunohistochemical detection of the neuromarker S100 protein (19) was negative in all aortic tunicae investigated, indicating the absence of nerve terminals in these vascular tissues (15). Another important piece of evidence for the existence of endothelial-derived catecholamines was the identification of the basal release of dopamine, noradrenaline, and adrenaline from tortoise isolated aortas detected by liquid chromatography coupled to tandem mass spectrometry (LC-MS/MS) and the significant reduction observed on the release when the endothelial cells were removed by mechanical procedure (20, 21).

To assess the potential modulatory role of endothelium-derived catecholamines in human vascular tissue, we have performed experiments in human umbilical cord vessels. Human umbilical cord vessels constitute a very interesting preparation for this particular purpose, since the umbilical cord has no innervation, as demonstrated by the absence of cholinergic and adrenergic nerve fibers, using fluorescence neurohistochemistry (22) and immunohistochemistry (23). The first clear demonstration that human endothelial cells could produce/release catecholamines was obtained using human isolated umbilical cord vessels (arteries and vein) in an organ bath and measuring catecholamine release by LC-MS/MS in the Krebs-Henseleit’s solution (24). Basal release of dopamine is present in human umbilical arteries and veins, and this release of dopamine was virtually abolished when the endothelial cells were taken away, indicating that the endothelium integrity was essential for dopamine release. The presence of tyrosine hydroxylase was confirmed by immunohistochemical methods, and tyrosine hydroxylase mRNA was detected by fluorescence in situ hybridization in the cytoplasm of the endothelial cells of the human umbilical cord vessels. As expected, immunohistochemistry for the neuromarker calretinin (25) was negative in the endothelia and in the tunic media of both vessels (24). The potential modulatory role was indicated by the findings that the contractions of human umbilical artery and vein rings (induced by electrical field stimulation) were insensitive to tetrodotoxin, significantly reduced by the α-adrenoceptor antagonist phentolamine, the dopamine D_2_-like antagonist haloperidol, and abolished by removal of the endothelial cells (24, 26).

## Endothelial-Derived Dopamine

Dopamine itself does not show contractile activity in the human umbilical artery rings unless the tissue is pretreated with the NO synthase inhibitor l-NAME (24). Although the canonical explanation for this observation is that dopamine causes the release of NO that will stimulate soluble guanylate cyclase and increase cGMP (27), an interesting alternative is that the production of NO will lead to the formation of 6-ND that will act as a dopamine antagonist and therefore will block the contractions induced by dopamine (to be developed in *Endothelial-Derived 6-Nitrodopamine*). Dopamine induces contractions in human umbilical vein rings, and the contractions were significantly increased in the vein rings that were pretreated with l-NAME (24). Dopamine has five receptors, and they are organized into two families: the dopamine D_1_-like receptor subtypes (D_1_R and D_5_R), associated with adenylyl cyclase stimulation, and the D_2_-like subfamily (D_2_R, D_3_R, and D_4_R), linked to inhibition of adenylyl cyclase (28). They are all present in vascular tissues, identified by radioligand-receptor binding and autoradiographic techniques (29) and immunohistochemical analysis (30). In both tissues, the contractions induced by dopamine were blocked when the rings were preincubated with the dopamine D_2_-like antagonist, haloperidol (24). Similar results were observed in tortoise aortic rings, where preincubation with either haloperidol or quetiapine caused significant rightward shifts in the concentration-dependent contractions induced by dopamine (21).

## Endothelial-Derived 6-Nitrodopamine

The first evidence of endogenous production of a nitrocatecholamine was generated by the observation that noradrenaline levels detected by microdyalisis of the rat hypothalamic paraventricular nucleus were decreased when the tissue was perfused with a solution containing nitric oxide (31). Using 6-nitronoradrenaline synthetized by bubbling NO gas in a solution containing noradrenaline hydrochloride as a standard, extracts from the porcine brain were analyzed by electrochemical detection linked to high-pressure chromatography, and a peak with an identical retention time to the standard was identified and characterized as 6-nitronoradrenaline by UV spectrometry, mass spectrometry (MS), and NMR spectroscopy (31). Employing the same methodological approach, the presence of 6-nitronoradrenaline was also detected in extracts from the rat brain, and the amounts of 6-nitronoradrenaline were significantly decreased (∼60–70%), but not abolished, in brain extracts obtained from rats previously treated with the NO synthase inhibitor l-NAME (31). Using the reaction of catecholamines with sodium nitrite to generate 6-nitronoradrenaline, 6-nitroadrenaline, and 6-nitrodopamine (32), the presence of 6-nitronoradrenaline and 6-nitronoradrenaline in extracts of rat brain was quantified by HPLC employing an online reducer column with platinum/rhodium-coated alumina fluorescence derivatization with ethylenediamine followed by chemiluminescence reaction detection (33). Basal release of 6-nitrodopamine (6-ND), measured by HPLC positive electrospray tandem mass spectroscopy (HPLC-MS/MS), with a limit of quantitation of 100 pg/mL, was identified from the following vascular tissues in vitro: human umbilical artery and vein (34), human popliteal arterial and vein (35), tortoise aorta (36), corn snake aorta (37), and pulmonary artery and aorta obtained from the marmoset *Callitrix s*pp. (109). The basal release of 6-ND from the above vascular tissues was significantly reduced, but not abolished, when the vascular tissues were pretreated (30 min) with l-NAME and virtually abolished when the endothelial cells were taken away. As reported for the aortic rings of venomous snakes, such as rattlesnake and *Bothrops jararaca*, as well as tortoise, detection of the neuromarkers S-100 protein and calretinin by immunohistochemical methods was also absent in corn snake aortic tunica intima and tunica media (15), excluding a neurogenic origin for 6-ND. Apart from vascular tissues, basal release of 6-ND has been observed from rat isolated right atrium (39), mouse isolated right atria and ventricles (38), rabbit isolated atria and ventricles (40), rat isolated vas deferens (41), and human isolated vas deferens (42).

## Actions of 6-Nitrodopamine in the Vascular Smooth Muscle

Contrary to dopamine, noradrenaline, and adrenaline, 6-ND was devoid of contractile action in human umbilical cord vessels, even when the vascular rings were preincubated with either l-NAME or 1H-[1,2,4]-oxadizaolo[4,3-]quinoxaline-1-one (ODQ). Preincubation of human umbilical cord vessels with 6-ND (10 µM) caused rightward shifts of the dopamine concentration-response curves (pA_2_ of 5.96 and 5.72, for human umbilical artery and vein, respectively), but it had no effect on the contractions induced by either noradrenaline or adrenaline (34). In human umbilical cord vessels, dopamine causes contractions via D_2_-like receptors, since the D_2_-like receptor antagonist haloperidol (43) abolished them. The finding that 6-ND also antagonized the contractions induced by the selective dopamine D_2_R agonist sumanirole (44) indicates that 6-ND in the umbilical cord vessels could act as a dopamine D_2_-like receptor antagonist. The selectivity of 6-ND for dopamine receptors is quite remarkable, since dopamine D_2_-like antagonists are promiscuous and bind to adrenergic receptors. For instance, the differences in potencies (*k*_i_) for D_2_ and α_1_-adrenoceptors for haloperidol (2.2 and 4.7 nM, respectively) and risperidone (2.2 and 1.4 nM, respectively) are very discrete (45, 46).

**FIGURE 1.**
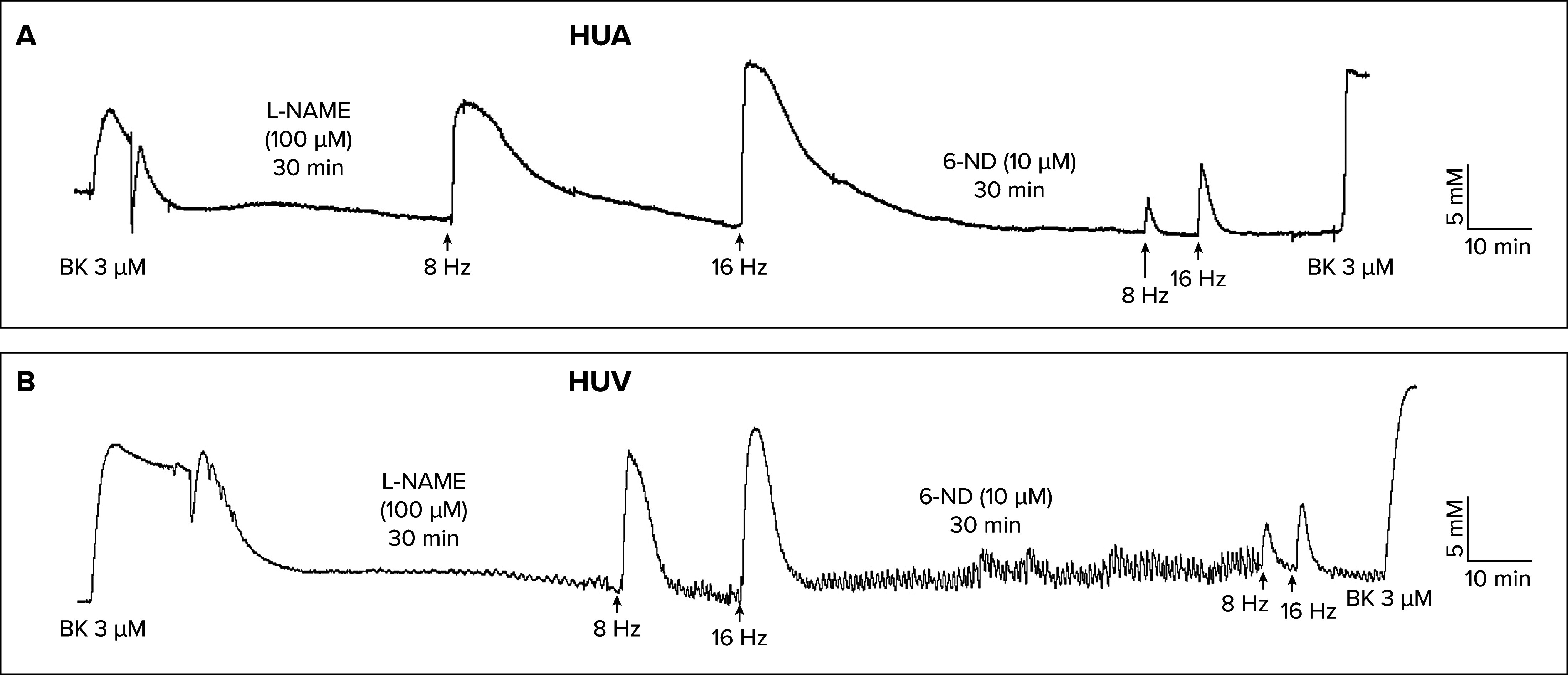
Representative trace of the incubation Representative trace of the incubation (30 min) of 6-nitrodopamine (6-ND; 10 µM) on the contractions induced by electrical field stimulation in human umbilical artery (HUA; *A*) and human umbilical vein (HUV; *B*) rings. BK, bradykinin; l-NAME, nitro-l-arginine methyl ester. Adapted from Ref. 34, with permission from *Life Sciences.*

**FIGURE 2.**
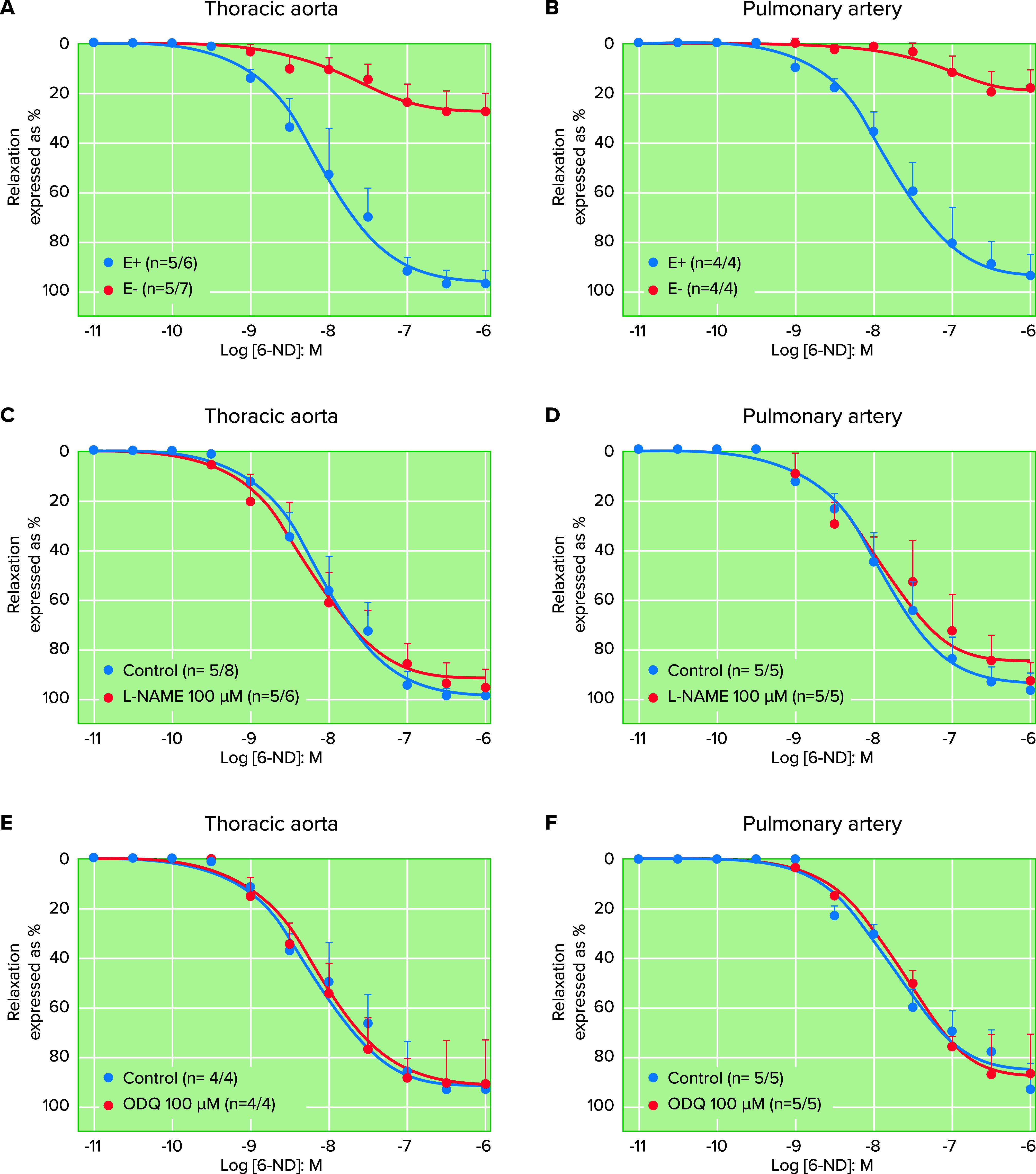
Concentration-dependent relaxations of the marmoset thoracic aorta (*A*, *C*, and *E*) and pulmonary artery rings 6-nitrodopamine (6-ND) causes concentration-dependent relaxations of thoracic aorta (*A*, *C*, and *E*) and pulmonary artery (*B*, *D*, and *F*) rings The rings were precontracted with the thromboxane mimetic U-46619 (3 nM). The relaxations induced by 6-ND were virtually abolished when the endothelium (E) was mechanically removed (*A* and *B*) but unaltered when the tissues are treated with either nitro-l-arginine methyl ester (l-NAME; *C* and *D*) or 1H-[1,2,4]-oxadizaolo[4,3-]quinoxaline-1-one (ODQ; *E* and *F*). The number of experiments was defined as *n* = *x*/*y*, where *x* represents the number of animals and *y* the number of rings employed. Adapted from Ref. 109, with permission from *Brazilian Journal of Medical and Biological Research*, per Open Access terms.

However, what is the possible physiological implication of this finding? The contractions provoked by electrical field stimulation in the human umbilical artery (FIGURE 1A) and vein (FIGURE 1B) were inhibited when the vascular rings were incubated with 6-ND, indicating that the contractile agent released by EFS was dopamine. Thus endothelial-derived dopamine plays an important role as a contractile agent and the balance between the release of dopamine and 6-ND could be an important factor in the modulation of vascular reactivity. It is important to note that 6-ND in vitro can act as a potent vasorelaxant agent; in the thoracic aorta and pulmonary artery rings that were precontracted with U-46619, the pEC_50_ was 8.1 and 7.8, respectively (38), whereas the pEC_50_ for acetylcholine in the isolated canine uterine artery was 6.9 (47). In marmoset thoracic aorta and pulmonary artery rings, which were precontracted with the endoperoxide analog U-46619, 6-ND caused concentration-dependent relaxations, which were virtually abolished when the endothelial cells were taken away (FIGURE 2, A AND B) but unaffected when the vascular rings were previously incubated with either l-NAME (FIGURE 2, C AND D) of ODQ (FIGURE 2, E AND F). Since the relaxation of the vascular smooth muscle is attributed to soluble guanylate cyclase and cGMP generation (48), and the relaxation induced by 6-ND was unaltered by soluble guanylyl cyclase inhibition, the synthesis of 6-ND constitutes an additional pathway by which NO causes vasorelaxation. Indeed, 6-nitrodopamine failed to increase cGMP levels in human washed platelets (49). The relaxation caused by 6-ND was not affected by pretreatment of the vascular rings with the dopamine D_1_R antagonist SCH-23390 (50), supporting the hypothesis that 6-ND is acting as a selective D_2_-like antagonist. Indeed, haloperidol also induced relaxation in U-46619 precontracted vascular rings, which was absent when the endothelial cells were removed (51) and not affected by either l-NAME or ODQ pretreatment.

**FIGURE 3.**
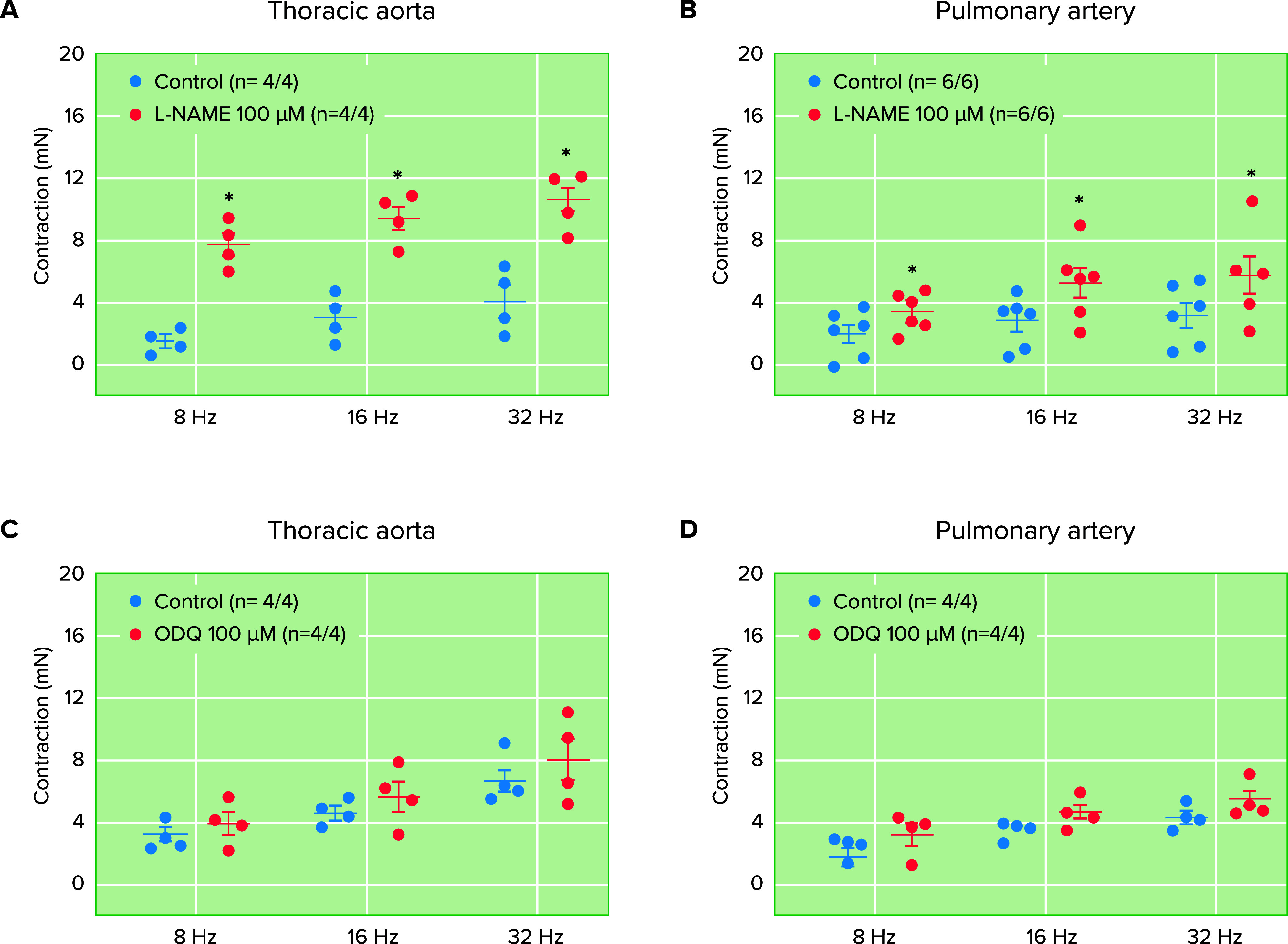
Effect of nitro-l-arginine methyl ester (l-NAME) and 1H-[1,2,4]-oxadizaolo[4,3-]quinoxaline-1-one (ODQ) on electrical field stimulation-induced marmoset thoracic aorta and pulmonary artery ring contractions Effect of l-NAME and ODQ on electrical field stimulation (EFS)-induced thoracic aorta (*A* and *C*) and pulmonary artery (*B* and *D*) ring contractions. Preincubation (30 min) with the nitric oxide synthase inhibitor l-NAME caused significant increases in frequency-dependent contractions induced by EFS in both thoracic aorta (*A*) and pulmonary artery (*B*) rings. In contrast, preincubation with the heme-site inhibitor of the nitric oxide-sensitive guanylyl cyclase ODQ did not affect the frequency-dependent contractions induced by EFS in either thoracic aorta (*C*) or pulmonary artery (*D*) rings. The number of experiments was defined *n* = *x*/*y*, where *x* represents the number of animals and *y* the number of rings employed. Data are reported as means ± SE. *P < 0.05 (Student’s unpaired *t* test). Adapted from Ref. 109, with permission from *Brazilian Journal of Medical and Biological Research*, per Open Access terms.

As observed in the brain extract of rats treated with the NO synthase inhibitor l-NAME (31), incubation of the tissues with l-NAME causes significant reductions (∼60–70%) in the basal release of 6-ND by human and reptile vascular tissues, indicating the existence of biosynthetic pathway(s) independent of NO synthase. For instance, endothelial cells produce nitrite ions (${\text{NO}}_{2}^{-}$) and hydrogen peroxide (H_2_O_2_) (52), and dopamine in phosphate buffer (pH 7.4) is oxidized to 6-ND by hydrogen peroxide-dependent enzymes such as horseradish peroxidase or lactoperoxidase in the presence of H_2_O_2_ and ${\text{NO}}_{2}^{-}$ (32). Thus the physiological role of 6-ND as a vasodilator may depend on both the vascular bed and the local conditions that may enhance or decrease dopamine nitration. This concept is clearly illustrated in experiments performed with the marmoset thoracic aorta and pulmonary artery rings. As reported for other vascular rings, preincubation with l-NAME significantly potentiated the contractions of the thoracic aorta (FIGURE 3A) and pulmonary artery (FIGURE 3B) rings induced by electrical field stimulation. However, no potentiation was observed when the thoracic aorta (FIGURE 3C) and pulmonary artery (FIGURE 3D) were pretreated with ODQ, demonstrating that in these vascular tissues the major mechanism by which NO attenuates vasoconstriction was the synthesis of 6-ND, rather than stimulation of soluble guanylate cyclase by NO (38).

## Actions of 6-Nitrodopamine in the Heart

**FIGURE 4.**
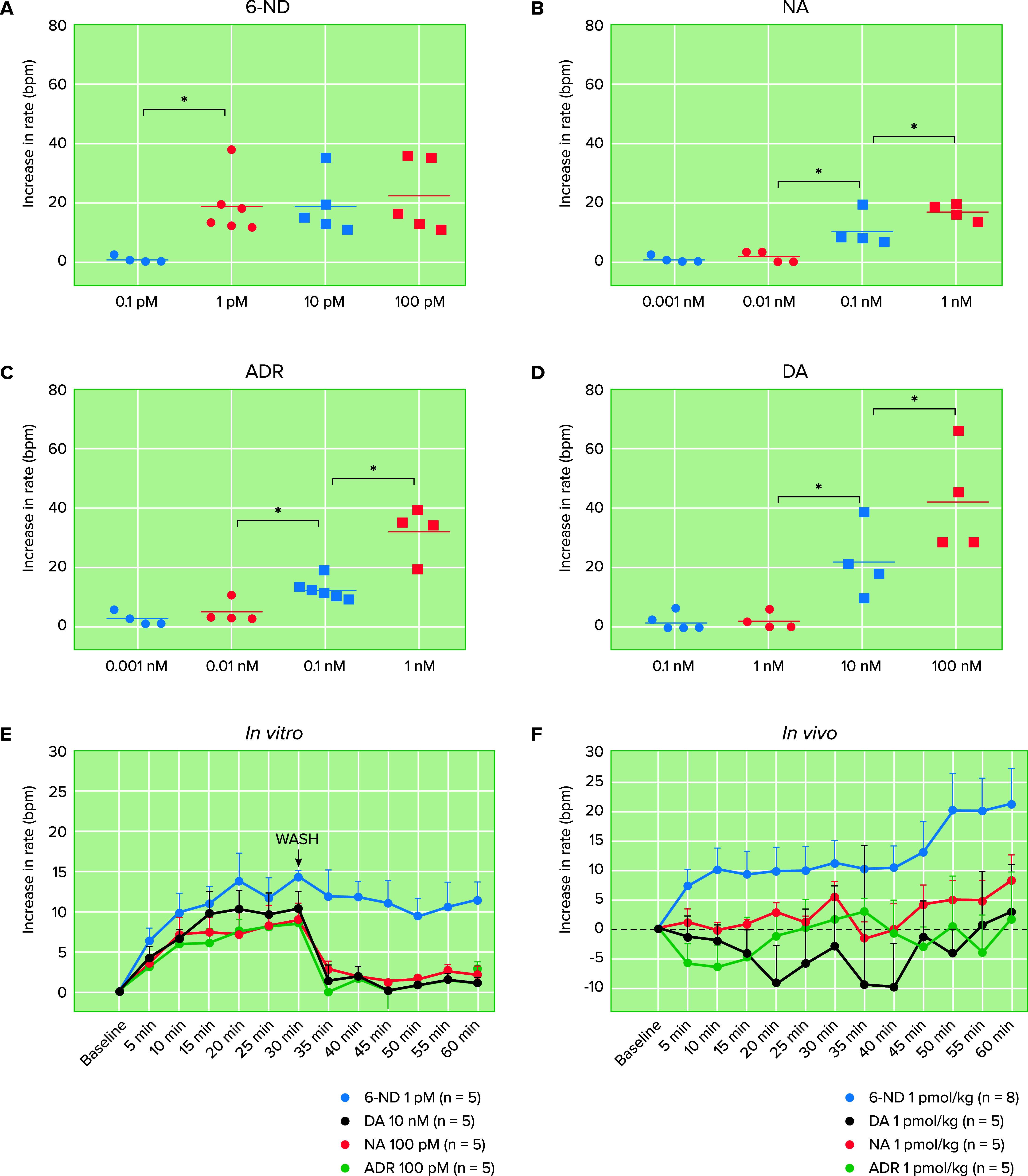
Concentration**-**response curves of catecholamines in the rat isolated right atrial rate Incubation (30 min) of the right atrium with l-NAME (100 µM) caused significant falls in atrial rate (*A*; bpm, beats/min), which were not observed when the right atrium was incubated (30 min) with ODQ (100 µM; *B*). In atrial pretreated with ODQ (100 µM), incubation with l-NAME (100 µM) provoked significant reductions in the atrial rate (*C*). Incubation of the right isolated atrial with atenolol provoked concentration-dependent (0.1 and 1 µM) falls in atrial rate (*D* and *E*). In atria pretreated with l-NAME (100 µM; *F*) or in atria obtained from animals chronically treated with l-NAME (*G*), atenolol (1 µM) did not cause a fall in atrial rate (*F* and *G*). *P < 0.05. Adapted from Ref. 39, with permission from *Life Sciences*.

Rat isolated right atrium releases higher amounts of 6-nitrodopamine compared to classical catecholamines, and the positive chronotropic effect induced by 6-ND (FIGURE 4A) was more potent (100 times) than noradrenaline (FIGURE 4B) and adrenaline (FIGURE 4C) and 10,000 times more potent than dopamine (FIGURE 4D). In contrast to the classical catecholamines, the positive chronotropic effect induced by 6-ND presented a unique characteristic; the increase in atrial rate was maintained even after washout of the agonist, whereas the augmentation of the atrial caused by the classical catecholamines would rapidly disappear after the exchange of the Krebs-Henseleit’s solution (FIGURE 4E). This prolonged action was also observed in the anesthetized rat (FIGURE 4F) (39). Another remarkable characteristic of 6-ND as a positive chronotropic agent is its ability to synergize with the classical catecholamines; at 1 pM, noradrenaline (FIGURE 4B), adrenaline (FIGURE 4C), or dopamine (FIGURE 4D) had no positive chronotropic effect on the rat isolated right atrium. 6-ND at 10 fM had no chronotropic effect on the rat isolated atrium (FIGURE 4A); however, when 10 fM of 6-ND was incubated with 1 pM of either dopamine, noradrenaline, or adrenaline, significant increases in atrial rate were observed, which persisted over 30 min after washout of the agonist (53). This extraordinary potentiation of 6-ND on the effects induced by noradrenaline, adrenaline, and dopamine has also been observed in the rat isolated vas deferens (110).

**FIGURE 5.**
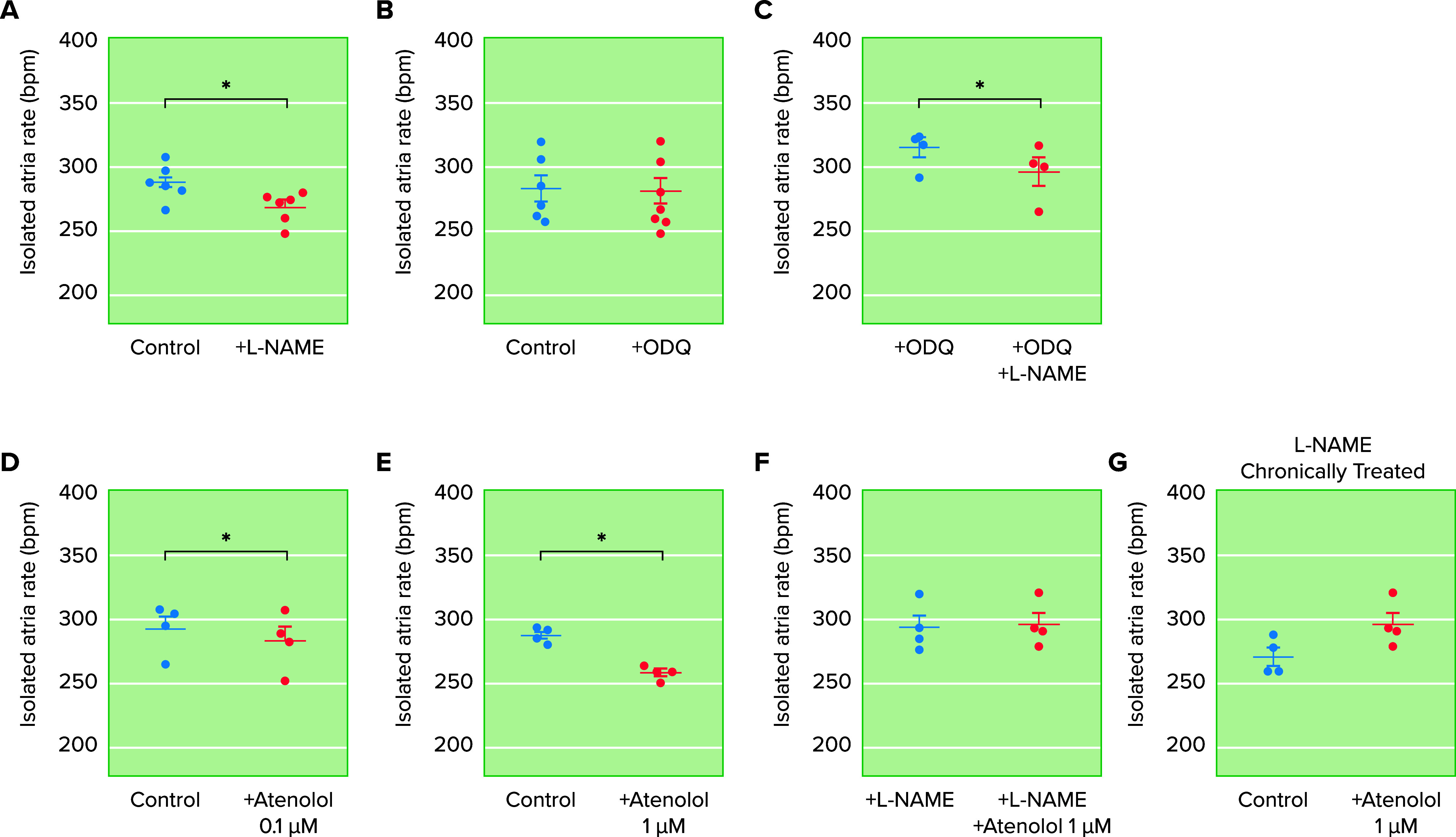
Effect of nitro-l-arginine methyl ester (l-NAME), 1H-[1,2,4]-oxadizaolo[4,3-]quinoxaline-1-one (ODQ), and atenolol on the basal rate of the rat isolated right atrium Incubation (30 min) of the right atrium with l-NAME (100 μM) caused significant falls in atrial rate (*A*; bpm, beats/min), which were not observed when the right atrium was incubated (30 min) with ODQ (100 μM; *B*). In atrial pretreated with ODQ (100 μM), incubation with l-NAME (100 μM) provoked significant reductions in the atrial rate (*C*). Incubation of the right isolated atrial with atenolol provoked concentration-dependent (0.1 and 1 µM) falls in atrial rate (*D* and *E*). In atria pretreated with l-NAME (100 μM; *F*) or in atria obtained from animals chronically treated with l-NAME (*G*), atenolol (1 μM) did not cause a fall in atrial rate (*F* and *G*). *P < 0.05. Adapted from Ref. 39, with permission from *Life Sciences*.

Having confirmed that rat atria release 6-ND and exogenous 6-ND increases atrial frequency, does endogenous 6-ND modulate atrial rate? Incubation of the rat isolated right atrium with l-NAME, resulted in significant inhibition of the basal release of 6-ND, indicating that the NO generated by NOS plays a role in the biosynthesis of 6-ND in the atria (39). Indeed, atria obtained from rats chronically treated with the l-NAME (54) also had a reduced basal release of 6-ND as compared to control animals. Inhibition of 6-ND release from the atria by incubation with l-NAME is associated with an important reduction in atrial frequency (FIGURE 5, A AND C), indicating that this fall could be a consequence of decreased endogenous release of 6-ND. In addition, the atrial rate observed in the atria obtained from rats chronically treated with l-NAME also has a decreased frequency compared to atria obtained from control animals (FIGURE 5C). It is important to mention that the increase in blood pressure generated by acute (55) or chronic (56) administration of l-NAME is associated with an important decrease in heart rate. This is not due to a cholinergic reflex induced by the increase in systemic blood pressure, since in other models of hypertension such as spontaneous hypertensive rats (57), two kidney one-clip (58), one kidney one-clip (59), and deoxycorticosterone acetate salt (60), the heart frequency was either augmented or unaltered. In addition, incubation of l-NAME with the atria results in a fall of atrial frequency even in atria pretreated with atropine (39). Indeed, in conscious rats pretreated with atropine, acute administration of l-NAME resulted in a notable decrease in heart rate (61), and in anesthetized dogs, a reduction in the heart rate was observed even when the vagi were sectioned (62). Fall in heart rate following administration of other NOS inhibitors such as monomethyl-l-arginine acetate and nitro-l-arginine has been reported in conscious dogs (63), rabbits (64), and sheep (65). In healthy volunteers, intravenous infusion (8 minutes) of l-NAME resulted in a dose-dependent (0.25, 0.5, and 0.75 mg/kg) fall in heart rate (66).

Stimulation of soluble guanylate cyclase is considered a classical mechanism of action of NO (48). However, in contrast to l-NAME (FIGURE 5A), incubation with ODQ, the heme-site inhibitor of nitric oxide-sensitive guanylyl cyclase (67), failed to alter atrial frequency (FIGURE 5B). In addition, incubation of l-NAME in atria pretreated with ODQ resulted in a fall in atrial rate (FIGURE 5C), indicating that this transduction mechanism may not be relevant for the modulation of heart chronotropism. In the sGCα1 knockout mouse, there was no difference in heart rate between sGCα1^−/−^ and wild-type mice (68). It is interesting that administration of the NO synthase inhibitor l-NAME increased the systemic blood pressure similarly in both wild-type and sGCα1^−/−^ mice, indicating that NO-sGC-cGMP pathway may not be essential in the modulation of vascular reactivity in vivo. It is worth noting that ODQ administration to rats caused no alterations in either mean arterial blood pressure or heart rate, although ex vivo inhibition of soluble guanylate cyclase was confirmed (69).

Thus, if 6-ND modulates heart chronotropism, what is(are) the mechanism(s) involved? The adrenergic nervous system is considered to play an essential role in regulating changes in the cardiovascular system; stimulation of the postganglionic sympathetic nerves causes noradrenaline release from the presynaptic nerve terminal located across the synaptic cleft from the cardiomyocyte (1). The released noradrenaline can bind to β_1_- and β_2_-postsynaptic adrenoceptors, but it can also bind to presynaptic β_2_- and α_2_-adrenoceptors, which can increase and decrease the rate of noradrenaline release, respectively. The β_1_-selective and β_1_β_2_-adrenoceptor antagonists are known to cause a reduction in the heart rate, both in vitro and in vivo, but is the mechanism through the blockade of β_1_-adrenoceptors or the 6-ND receptor? In the rat isolated right atrium, the β_1_-selective adrenoceptor antagonist atenolol, at 100 nM, inhibited the positive chronotropic effect induced by 1 pM of 6-ND, without affecting the positive chronotropic effect induced by 10 nM of dopamine, or by 100 pM of either noradrenaline or adrenaline. Similar results were observed with the β_1_-selective adrenoceptor antagonists betaxolol and metoprolol at 100 nM (34). These results demonstrate that the β_1_-selective adrenoceptor antagonists are more potent inhibitors of the action of 6-ND than the classical catecholamines. Atenolol provoked a concentration-dependent inhibition of the 6-ND positive chronotropic effect (FIGURE 5, D AND E); however, the inhibition was absent when the atria were previously incubated with l-NAME (FIGURE 5F) or when the atria were obtained from animals chronically treated with l-NAME (FIGURE 5G). Similar results were also observed with atria treated with the other β_1_-adrenoceptor antagonists betaxolol or metoprolol (34). Thus the negative chronotropic effect induced by the so-called β_1_-adrenoceptor antagonists is due to inhibition of 6-ND action rather than actions induced by the classical catecholamines. Indeed, in atria pretreated with the sodium channel blocker tetrodotoxin, the β_1_-adrenoceptor antagonists still caused notable reductions in the atrial frequency, whereas atropine failed to increase atrial frequency, indicating that autonomic nerve terminals were inhibited by tetrodotoxin (34).

**FIGURE 6.**
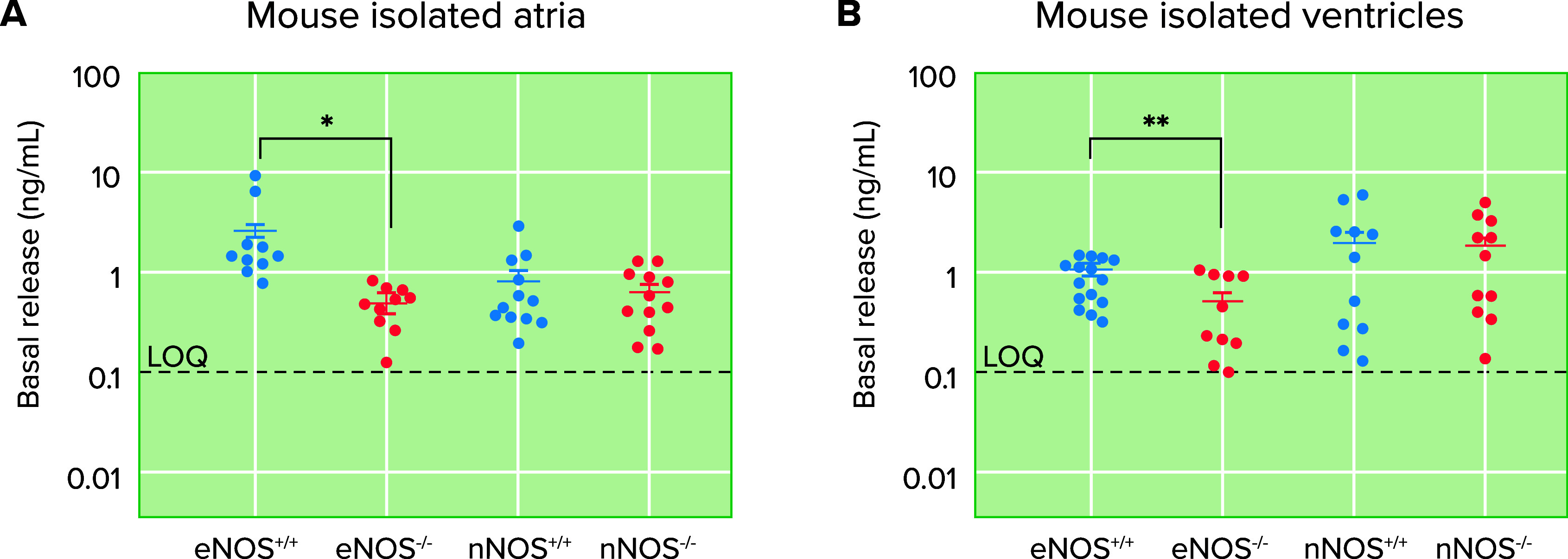
Basal release of 6-nitrodopamine (6-ND) from mouse isolated right atria and ventricles Basal release of 6-ND in isolated right atria (*A*) and ventricles (*B*) obtained from eNOS^+/+^ (control), eNOS^−/−^, nNOS^−/−^ (control), and nNOS^−/−^ mice. 6-ND was measured by liquid chromatography coupled to mass spectrometry. eNOS, endothelial nitric oxide synthase; nNOS, neuronal nitric oxide synthase; LQQ, limit of quantification. Significant difference: *P < 0.05; **P < 0.01. Adapted from Ref. 38, with permission from *Nitric Oxide*.

Is 6-ND released in the atria neurogenic or endothelium derived? The result that incubation of the rat isolated atria with tetrodotoxin did not alter the basal release suggests that it is unlikely that 6-ND originates in nerve terminals. Mouse isolated atria (FIGURE 6A) and ventricles (FIGURE 6B) show 6-ND release, and this basal release is significantly decreased in endothelial nitric oxide synthase knockout (eNOS^−/−^) mice (FIGURE 6, A AND B) and unaffected in neuronal nitric oxide synthase knockout (eNOS^−/−^) mice (FIGURE 6, A AND B), indicating a major role for eNOS on the biosynthesis of the 6-ND in the heart. Interestingly, the atrial frequency observed in eNOS^−/−^ mice is significantly reduced when compared to that observed in atria obtained from control animals, whereas that observed in atria obtained from nNOS^−/−^ mice is unaltered. In addition, eNOS^−/−^ mice have a lower heart rate as compared to control animals (70), whereas no difference in vivo in the basal heart frequency from nNOS^−/−^ and wild type has been observed (71). Thus eNOS-derived 6-ND is apparently the major endogenous modulator of heart chronotropism.

Another important feature of 6-ND is its ability to act as a positive inotropic agent. In the rat isolated perfused heart (Langendorff’s preparation), 6-ND was the major catecholamine released from the ventricles, and it was 1,000 times more potent than dopamine and noradrenaline, and 10,000 times more potent than adrenaline, as a positive inotropic drug. Interestingly, the positive inotropic effect of 6-ND was inhibited by the supposed β_1_-adrenoceptor antagonist atenolol at a concentration (10 nM) that did not affect the positive inotropic effect induced by dopamine, noradrenaline, and adrenaline. At this concentration, atenolol caused a significant reduction of the basal left ventricular developed pressure, indicating that it may be acting as a selective 6-ND receptor antagonist, rather than a β_1_-adrenoceptor antagonist (72).

## Novel Developments

**FIGURE 7.**
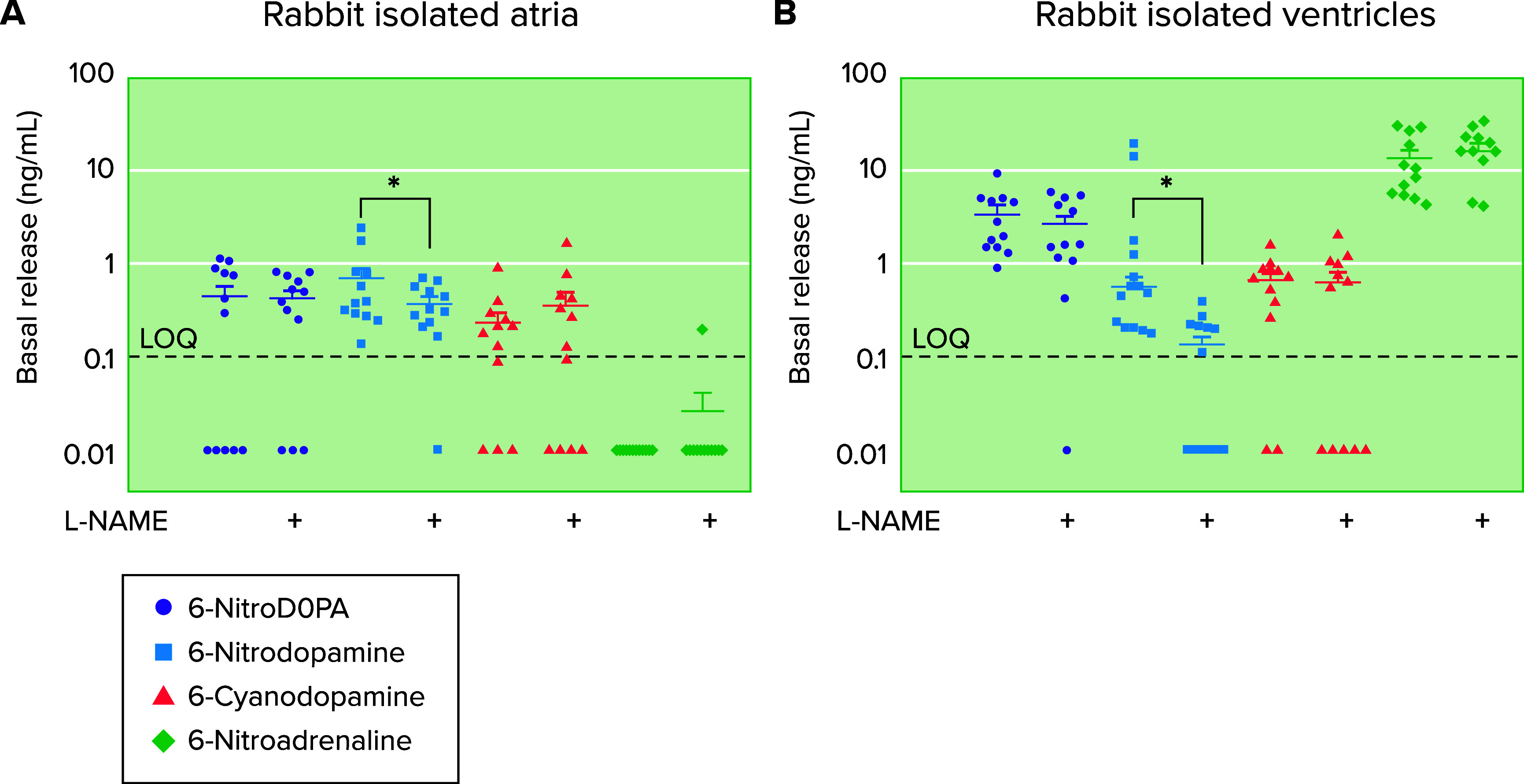
Basal release of 6-nitrodopa, 6-nitrodopamine, 6-cyanodopamine and 6-nitroadrenaline from rabbit isolated atria (*A*) and ventricles. Basal release of 6-nitrodopa, 6-nitrodopamine, 6-cyanodopamine, and 6-nitroadrenaline from rabbit isolated atria (*A*) and ventricles (*B*) The catecholamines were measured by liquid chromatography coupled to mass spectrometry. LQQ, limit of quantification. + Indicates that the heart chambers were preincubated (30 min) with nitro-l-arginine methyl ester (l-NAME) (100 µM). *P < 0.05. Adapted from Ref. 41, with permission from *Biomedical Chromatography*.

The identification of basal release of other novel catecholamines will expand our understanding of the physiology and physiopathology of the cardiovascular system. Basal release of 6-nitrodopa, 6-nitrodopamine and 6-cyanodopamine was detected by a fully validated high-pressure chromatography positive electrospray linked to tandem spectroscopy (LC-ESI-MS/MS) (40) from rabbit isolated atria (FIGURE 7A). The release of 6-nitroadrenaline was not detected (FIGURE 7A). Rabbit isolated ventricles also basally released 6-nitrodopa (FIGURE 7B), 6-nitrodopamine (FIGURE 7B), 6-cyanodopamine (FIGURE 7B), and 6-nitroadrenaline (FIGURE 7B). Note that 6-nitroadrenaline was not identified in the basal release from atria, yet it was by far the major (10 times higher levels) catecholamine released from the ventricles. The discovery of this new class of endogenous catecholamines, the 6-cyano-catecholamines, indicates the possibility of the existence of other classes of endogenous catecholamines (6-bromo-catecholamines, etc.), with possible distinct cardiovascular actions.

**FIGURE 8.**
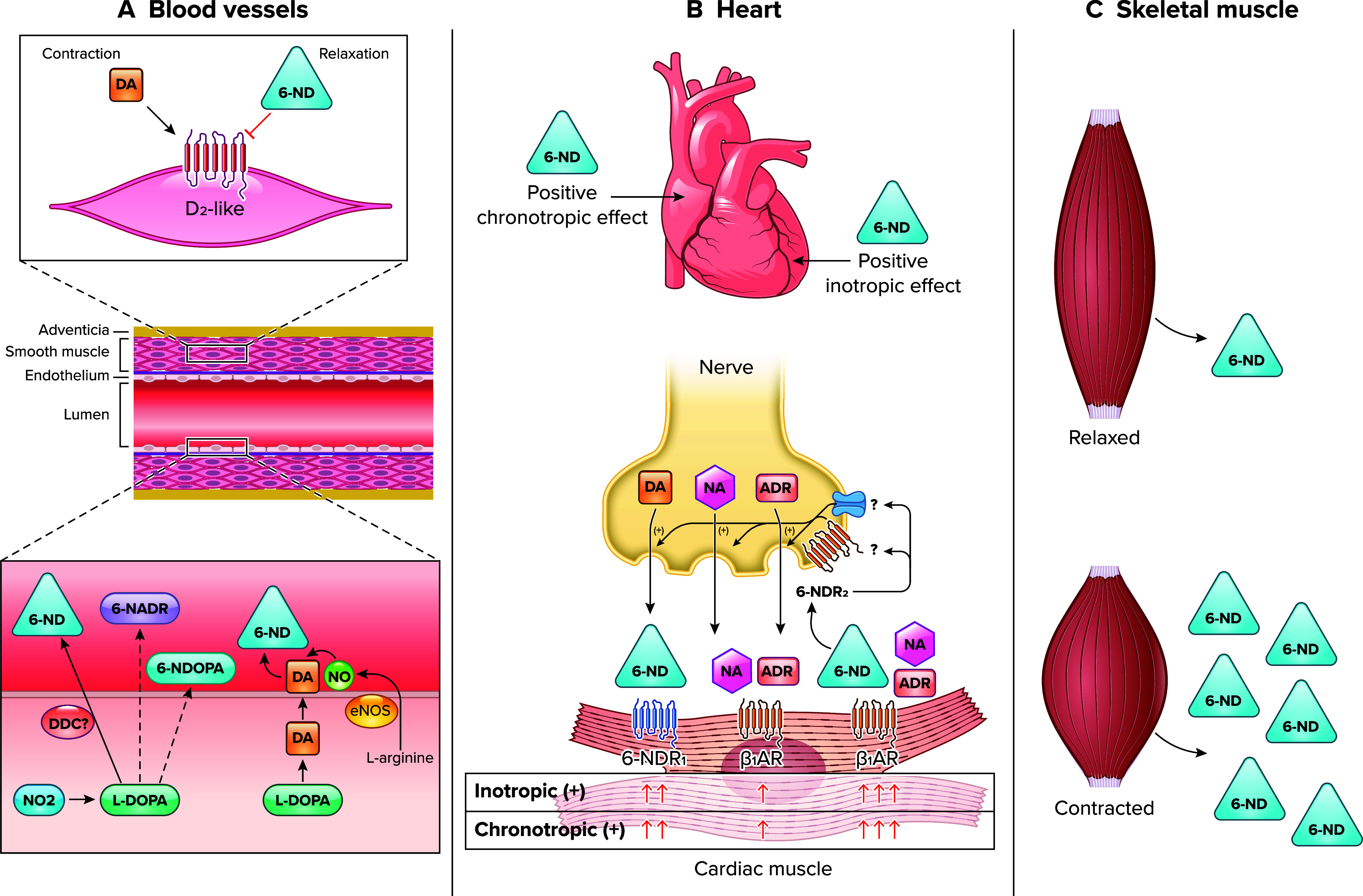
Release and action of endothelium-derived dopamine and 6-nitrodopamine (6-ND) in the cardiovascular system *A*: nitric oxide synthase (NOS)-dependent and NOS-independent pathways for 6-ND biosynthesis and the mechanism for its vasodilator action. *B*: mechanisms involved in the positive chronotropic and inotropic effects induced by 6-ND. *C*: hypothesis that skeletal muscle releases 6-ND and that contractions of the skeletal muscle may cause an increase in the basal release of 6-ND, as a possible mechanism to avoid orthostatic hypotension. DA, dopamine; ADR, adrenaline; NA, noradrenaline; NO, nitric oxide; eNOS, endothelial nitric oxide synthase; 6-NDOPA, 6-nitrodopa; 6-NADR; 6-nitroadrenaline; l-DOPA, l-3,4-dihydroxyphenylalanine; D_1_R, D_1_-like receptor; β_1_AR, β_1_, adrenergic receptor.

The major biosynthetic pathway for 6-ND is dependent on the action of nitric oxide synthase to generate NO for the nitration of dopamine. This pathway occurs in the membrane of the endothelial cell, where it is located eNOS (FIGURE 8A). The finding that in rabbit isolated heart preincubation of the tissues with l-NAME caused significant reductions in the basal release of 6-nitrodopamine, whereas the basal release of either 6-nitrodopa or 6-nitroadrenaline was unaffected (40), indicates the existence of a NOS-independent biosynthetic pathway. One possibility is the reduction of endogenous nitrite (NO_2_) pools, generating nitrocatecholamines, which are released into the circulation (FIGURE 8A). Whether these nitrocatecholamines are stored in the heart remains to be investigated.

How does endothelium-derived dopamine and 6-nitrodopamine control the circulation? In the vasculature, 6-ND is devoid of contractile activity; however, it acts as a truly selective dopamine D_2-like_ receptor antagonist, causing vasodilatation (FIGURE 8A). In the heart, 6-ND presents both potent positive chronotropic and inotropic effects (FIGURE 8B); the evidence indicates the existence of two receptors for 6-ND in the heart, one located in the cardiomyocytes (6-ND1R) and the other in the adrenergic terminals (6-ND2R; FIGURE 8B). The former is responsible for the direct chronotropic/inotropic effect of 6-ND, whereas the latter for the remarkable synergism that 6-ND exhibits with the classical catecholamines.

Can these nonneurogenic mediators avoid orthostatic hypotension? As it is known, orthostatic hypotension, also known as postural hypotension, may occur when standing after sitting or lying down. The current dogma is that activation of the adrenergic nervous system causes vasoconstriction and an increase in heart chronotropism and inotropism to avoid hypotension. This is not a very “intelligent” mechanism, since it does not really prevent hypotension (you need to have a fall in blood pressure to activate the adrenergic nervous system). If this were true, patients with heart or heart-lung transplantation would be having orthostatic hypotension every time they would stand up. As shown in FIGURE 8C, one possibility is that 6-ND is also released from skeletal muscle. Before you stand up, you need to contract the skeletal muscles, and this would cause an increase in 6-ND release, which would have both positive inotropic and chronotropic effects on the heart. This idea that skeletal muscle controls the cardiac output is not original (73).

## Reappraisal of the Physiological Role of the Adrenergic Nervous System in the Heart and Kidney

The discovery of endothelium-derived catecholamines should promote a careful reassessment of the conclusions based on these two premises. The microcirculation modulates the vascular tone to adjust local tissue perfusion with oxygen and metabolic demands, yet there is neither adrenergic nor cholinergic innervation in the microcirculation. Local factors, mainly endothelial-derived catecholamines, should be considered now as major candidates for modulating vascular tone in the microcirculation. Although the heart presents extensive autonomic innervation, there are some pieces of evidence that the heart can perform well without innervation. In pediatric patients who underwent heart transplantation, resting heart rate was significantly higher in transplanted patients as compared to healthy controls (74). Pediatric heart transplant recipients may present either a near-normal rise in heart rate at exercise onset or an impaired change in heart rate at exercise onset (75), indicating that the presence of heart adrenergic innervation is not essential for increasing cardiac frequency and output. In adult patients who have received heart transplantation and submitted to a graded treadmill exercise, peak values (normal vs. denervated) for systolic blood pressure (189 vs. 167 mmHg), heart rate (172 vs. 159 beats/min), and work time (26.2 vs. 18.0 min) were increased in normal than in cardiac transplant recipients; however, those denervated heart patients did show a good response following exercise (76). A clinical investigation, comparing eight healthy heart-lung transplant recipients with eight normal subjects matched for age and sex, has shown that the former group had significantly higher diastolic blood pressure and heart rate (77). In addition, the increase of diastolic pressure and heart rate during head-up tilt presented no significant differences in the two groups. There were no significant differences in the basal circulating levels of noradrenaline and adrenaline in the two groups; however, during head-up tilt, there was a higher and significant increase in the noradrenaline circulating levels in the transplant group as compared to the control group. Since the patients submitted to heart-lung transplantation have the heart denervated, the endothelium and not the adrenal medulla should be the origin of those catecholamines.

Organ innervation is generally regarded as an important factor in the regulation of renal hemodynamics and key functional activities such as glomerular filtration rate (GFR) and sodium homeostasis (78–80). However, this concept has been recently challenged by numerous clinical and experimental observations. It is well known that autoregulation of GFR is entirely governed by local factors such as afferent arteriole myogenic reflex and/or tubuloglomerular feedback signaling (81). The fundamental pressure natriuresis property is conserved in isolated perfused kidneys (82). Decades of clinical experience have shown that transplanted kidneys are still capable of maintaining homeostasis and regulating blood pressure as well as renal hemodynamics, even though renal innervation has been completely severed. Experimental evidence obtained with renal cross transplantation has consistently shown that the blood pressure level is governed by the transplanted kidney, with hypertensive recipients becoming normotensive when receiving a kidney from a normotensive donor and vice versa (83, 84). Thus the kidneys can perform hemodynamic regulation independently of direct innervation, largely because relevant afferent signals and/or efferent stimuli can travel through the circulation. However, adrenalectomized rats, with no circulating catecholamines, are still capable of regulating sodium and potassium homeostasis through the action of endogenous vasopressin and/or exogenous aldosterone (85, 86). Even isolated kidney preparations can respond to stimuli such as perfusion pressure (pressure natriuresis). Together, these observations indicate that although nervous stimuli and blood-borne substances can modulate renal function, the bulk of renal regulatory activity must occur at the local level. This can only be accomplished through the local synthesis of vasoactive compounds.

Renal cells are capable of synthesizing a myriad of vasoactive substances that can act as local regulators of renal microcirculation. Given their potent effects on microvessels and sodium conservation, catecholamines deserve special attention. Early studies demonstrated that isolated rat kidneys can synthesize epinephrine, norepinephrine, and dopamine (87). Renal tubular cells are able to produce large amounts of dopamine, whereas cultivated glomerular mesangial cells can synthesize epinephrine, norepinephrine and dopamine (88), all of which may exert a key role in the renal processing of sodium. The renal microcirculation is an obvious target of locally produced substances, especially catecholamines. The renal endothelium is well known to synthesize and release a wealth of vasoactive compounds, such as vasodilating and vasoconstrictor prostaglandins, endothelin, and sulfidric gas (89). Direct evidence that renal endothelial cells release catecholamines or express the enzymes involved in their biosynthesis is currently lacking, although this capability has been shown for mesangial cells that can synthesize epinephrine, norepinephrine, and dopamine (88). However, evidence that these compounds are synthesized and released by endothelial cells of other territories has been obtained in mice, bovine aorta, and human umbilical cord vessels (16, 24), a finding compatible with the notion that the vascular endothelium can participate in the local regulation of the microcirculation. It is more than likely that the machinery for the biosynthesis of catecholamines is also present in renal endothelial cells. Endothelium-derived nitric oxide (NO), described in early studies as an endothelium-derived relaxing factor, has been amply shown to exert a strong, continuous vasodilating effect on renal microcirculation (89). In addition, NO has been extensively shown to enhance urinary sodium excretion (90). Acute inhibition of NO synthesis leads to marked systemic hypertension and renal vasoconstriction, with predominance of efferent vasoconstriction and sharp elevation of glomerular hydraulic pressure (91). Chronic NO inhibition promotes similar changes, along with severe glomerular and interstitial injury (54, 92).

NO is a short-lived molecule, with very limited ability to exert direct influence away from its site of production. In this context, the fact that endothelial cells developed a sophisticated machinery to synthesize NO underlines the importance of this molecule as a local regulator of the microcirculation. In the particular case of the kidneys, the intense effect of locally generated NO on microvessels is a perfect example of how the kidneys can regulate their own microcirculation, while keeping sodium homeostasis and blood pressure levels, independently of external factors. The influence of NO on renal function transcends its direct effect on microvessels. By acting on the glomerular microcirculation, particularly on the afferent arteriole, NO participates actively in the autoregulation of GFR (81, 93, 94). Additional effects of NO on this process are likely to occur at the macula densa, where neuronal NO synthase was extensively shown to be expressed (95, 96). Moreover, NO acts in the medullary circulation (97) and can thus influence the renal ability to concentrate the urine.

In early studies, the vasodilatory effect of NO was shown to be mediated by the formation of cyclic GMP (cGMP), known to cause direct relaxation of vascular smooth muscle cells (98, 99). In subsequent studies, NO was shown to promote nitration of intracellular proteins, which causes profound structural and functional changes (100, 101). Given the fact that, besides generating an enormous amount of NO, the renal endothelium in all likelihood synthesizes catecholamines, the possibility arises that nitration of the latter results in the formation of nitrocompounds such as 6-nitrodopamine and nitroadrenaline. This scenario is consistent with the recent finding that endothelial cells from human umbilical cord vessels and from the popliteal artery can release 6-nitrodopamine (34). Taken together, these observations reinforce the notion that the local synthesis of vasoactive compounds underlies the ability of the kidneys to maintain a regulatory activity independently of external nervous or humoral stimuli and raise the exciting possibility that the newly described endothelium-derived catecholamines have relevant participation in this process. The discovery of these compounds can not only have profound implications for cardiovascular and renal physiology and pathophysiology but can also significantly influence clinical practice: measurement of their circulating concentrations may yield important information on the condition of the heart and vessels; urinary output (and renal clearance) of endothelium-derived catecholamines and/or their metabolites may contribute to refining the evaluation of the functional status of the kidneys and of their response to conditions that affect the circulation; determination of their concentration in the dialysate effluent may be helpful in the assessment of patients undergoing peritoneal dialysis; endothelium-derived catecholamines may also have therapeutic applications in the management of arterial hypertension and of circulatory shock.

## Clinical Significance and Future Directions

Acute decompensated heart failure is a complex and life-threatening clinical syndrome, associated with recurrent hospitalizations and a high mortality rate (102). In hospitals, mortality ranges from 4 to 6% in the United States (103) up to 9% in the United Kingdom (104). The risk of mortality rises after hospital discharge reaching 10% at 30 days and 22–27% at 1 year (105). The initial clinical target is decongestion and hemodynamic stabilization, and intravenous loop diuretics such as furosemide remain the mainstay of pharmacological therapy (106). Although in patients presenting with acute heart failure and pulmonary edema without significant hypotension the guidelines recommend the use of vasodilators such as nitroglycerin and nitroprusside, evidence that these drugs are beneficial is lacking (107). Positive inotropes such as dobutamine, dopamine, and other adrenoceptors are recommended in patients with acute heart failure and evidence of end-organ hypoperfusion, but those drugs present dose-limiting adverse effects including atrial and ventricular arrhythmias and do not alter mortality outcome. In this context, the finding that 6-nitrodopamine is a very potent chronotropic and inotropic agent presents a remarkable synergism with the classical catecholamines in the cardiovascular system, and it is a potent vasodilator, offers an exciting therapeutic potential for not only acute heart failure but also for chronic heart failure. Although the pharmacokinetics of 6-nitrodopamine is not known, its effect on the heart both in vitro and in vivo is quite prolonged, which is advantageous compared to the classical catecholamines. Studies on the receptor characterization, biosynthesis, and metabolism of 6-nitrodopamine should allow us to better design clinical protocols to evaluate its therapeutic effect.

Besides the potential therapeutic effects, the identification of these novel catecholamines may be useful as biomarkers of pathophysiological processes. For instance, catecholamine plasma levels have no clinical importance, except for the diagnosis of pheochromocytoma (108), which is not a cardiovascular disease. Measurement of circulating levels and identification of novel urinary metabolites such as 6-nitro-homovanillic acid should help to further understand the role of the endothelium-derived catecholamines.
